# Backward neural network (BNN) based multilevel control for enhancing the quality of an islanded RES DC microgrid under variable communication network

**DOI:** 10.1016/j.heliyon.2024.e32646

**Published:** 2024-06-14

**Authors:** Hira Anum, Muntazim Abbas Hashmi, Muhammad Umair Shahid, Hafiz Mudassir Munir, Muhammad Irfan, A.S. Veerendra, Mohammad Kanan, Aymen Flah

**Affiliations:** a Institute of Mathematics, Khwaja Fareed University of Engineering & Information Technology (KFUEIT), Rahim Yar Khan, Pakistan; b Department of Electrical and Bio-medical Engineering, Khwaja Fareed University of Engineering & Information Technology (KFUEIT), Rahim Yar Khan, Pakistan; c Department of Electrical Engineering, Sukkur IBA University, Sukkur, 65200, Pakistan; d Department of Electrical and Electronics Engineering, Manipal Institute of Technology, Manipal Academy of Higher Education, Manipal, 576104, India; e Department of Industrial Engineering, College of Engineering, University of Business and Technology, Jeddah, 21448, Saudi Arabia; f Energy Processes Environment and Electrical Systems Unit, National Engineering School of Gabes, University of Gabes, Gabès, 6029, Tunis, Tunisia; g MEU Research Unit, Middle East University, Amman, 11831, Jordan; h Private Higher School of Applied Sciences and Technology of Gabes, University of Gabes, Gabès, 6029, Tunisia; i ENET Centre, VSB—Technical University of Ostrava, 708 00, Ostrava, Czech Republic; j Applied Science Research Center, Applied Science Private University, Amman, 11931, Jordan

**Keywords:** Renewable energy sources, Backward NN, NN microgrid control, Communication latencies, Multi-level control, Distributed control

## Abstract

Microgrids (MGs) and energy communities have been widely implemented, leading to the participation of multiple stakeholders in distribution networks. Insufficient information infrastructure, particularly in rural distribution networks, is leading to a growing number of operational blind areas in distribution networks. An optimization challenge is addressed in multi-feeder microgrid systems to handle load sharing and voltage management by implementing a backward neural network (BNN) as a robust control approach. The control technique consists of a neural network that optimizes the control strategy to calculate the operating directions for each distributed generating point. Neural networks improve control during communication connectivity issues to ensure the computation of operational directions. Traditional control of DC microgrids is susceptible to communication link delays. The proposed BNN technique can be expanded to encompass the entire multi-feeder network for precise load distribution and voltage management. The BNN results are achieved through mathematical analysis of different load conditions and uncertain line characteristics in a radial network of a multi-feeder microgrid, demonstrating the effectiveness of the proposed approach. The proposed BNN technique is more effective than conventional control in accurately distributing the load and regulating the feeder voltage, especially during communication failure.

## 1 Introduction

The distribution system of a DC microgrid (MG) gives a simple and feasible solution to several difficulties as compared to the distribution system of an AC microgrid (MG), which has frequency synchronization, complicated reactive power flow control, and inrush transformer currents problems [1,2]. In the last years, the viable transformation of DC is possible due to the growth of semi-conductor deceives [[3], [4], [5], [6], [7]]. A robust distributed secondary control (DSC) system is utilized for inverter-based microgrids (MGs) connected in sparse networks with uncertain communication. Through iterative learning mechanics, two discrete-time DSC controllers are designed. These controllers enable all DERs in the MG to achieve voltage/frequency restoration and accurate active power sharing [8,9]. However, the secondary control inputs are updated only at the end of each iteration round, reducing the need for DERs to exchange information with neighbors in a low-bandwidth manner. Before then, the power grid underwent significant advancement due to the integration of a more widely distributed generation system installed at the customer's location [10,11]. This finishes by emphasizing the need to establish a more efficient and cost-effective method. It aids in resolving the intricate power system that makes it more difficult to control, manage, and organize the network. A distributed network of the described DC MG is created to autonomously operate in a smaller territory than the main power network, using RES (Renewable energy source). The problem is to devise a viable infrastructure that can efficiently distribute the demand in conjunction with the existing power grid [12]. The current grid network consists of RES plus conventional power production components and loads which makes the network complex [[13], [14], [15], [16]].

The proposed approach combines optimal power flow and voltage-var optimization in a hybrid control system to satisfy the demand for the load, reduce transmission line losses, and keep the voltage within a useful range [17]. To accomplish this, a distributed neural network method is utilized to determine the most efficient solution for the flow of active power, hence decreasing the cost of active power [18,19]. A novel approach for secondary voltage control in islanded microgrids has been developed, utilizing fuzzy and adaptive control techniques [20,21]. This method incorporates the rules of the back-stepping method to generate a control signal. Additionally, the adaptive method provides fuzzy approximation coefficients [22]. A robust decentralized control method has been implemented within the islanded microgrid [23,24]. As a result of the increased adoption of energy storage devices and electric vehicles, system designers are increasingly opting for renewable energy source-based DC microgrids at the distribution level. Furthermore, as the majority of market loads consume direct current (DC) at the consumer level, microgrids employ streamlined control techniques for their functioning [25,26]. Recently, researchers have been working on the RES-based DC MG controller scheme for accomplishing the essential operation. For hierarchical control, RES-based DC MGs involve multilevel control such as centralized, decentralized, and distributed control schemes. Multi-level control is made of three levels one is primary, the second level is secondary, and the last one is tertiary level controls [20,[27], [28], [29]]. Each node uses a localized droop framework for primary control, which achieves the necessary load sharing by regulating the droop's virtual resistance (VR) to maintain the nodes' dynamic stability [30,31].

On a large-scale integration of the RES network, each node is requested to track common reference operating directions which are set by higher-level control. In contrast with the network, the node works for the local droop framework. Secondary control constructs the outer loop of the network, which is on the higher side of primary control. On the secondary level, each node shares its value with the neighbors for observation and converges to the joint operating point of the network to keep the MG smooth operation and stability [[32], [33], [34], [35], [36], [37]]. Secondary-level and tertiary-level controls are applied in a decentralized or centralized style in which they attach with the neighboring nodes utilizing the communication lines. Some uncertainty or failure may have led the single node to become a failure and instability of another node which may further lead to the possibility of a cascaded failure [[38], [39], [40], [41], [42], [43]]. Later it increases the complexity of the system w.r.t scalability and centralized control, a smart option for better and humble communication network topology is a distributed control scheme that reduces the price. Secondary control keeps informing the MG network about the set point using the small bandwidth communication system on each distributed energy resource [17,44]. Techniques of matrix theory and graph theory are used for system stability and robustness in contradiction to uncertainty.

The communication system of the RES-based DC MG has slow communication issues because it takes time to transfer information to the neighbors. Communication latencies or breaks can affect the control response to load imbalances and network stability [31]. The network would respond to delays by modifying the operation points in time for each node [[45], [46], [47]]. When a bi-directional communication network is used to transfer a large volume of data by utilizing a communication channel, communication errors can occur and affect MG performance [[48], [49], [50]]. The communication network serves the DC microgrid (MG) as a foundation of the network. By utilizing the communication network, the system can achieve the MG operation. Microgrids need quicker and more robust control due to the erratic nature of RE-based distributed generation units (DGUs) [[51], [52], [53], [54]]. Due to the discrepancy in operating communication links, reference nodes may experience overstress as a result of a loss in an unpredictable device. An excessively burdened node can lead to system instability and a chain reaction of failures. When faced with a communication breakdown, researchers initially employed a straightforward method to fix a device by disconnecting the secondary control and transitioning the network to a constant reference [55,56]. The key disadvantage of constant reference control is that each agent has a unique constant reference. This method of constant reference is often restricted to ring-connected power.

More robust transmission networks are typically used for better communication efficiency. To increase the robustness of the DC microgrid (MG) control structure, a backward neural network (BNN) is used at the secondary level. Conventional control cannot maintain the operation of DC MG during communication failure or latencies. BNN helps to maintain the operation during communication delays on the last operating directions shared. BNN shows an adaptive nature, which makes it resilient and robust. During a communication delay, the conventional consensus system does not maintain its working, operation, and stability. However, communication link failure may occur at any point in the network. So, the secondary control system of multi-level control is connected to the conventional communication network. To overcome this problem a secondary control system is proposed by utilizing a backward neural network (BNN). BNN is not sensitive to communication delays or latencies which helps to increase the robustness of DC MG control in maintaining MG's stability, working, and operation. Whereas, the conventional consensus system cannot withstand communication link failure or communication latencies. In this paper, BNN will be used to communicate between nodes to maintain the system's operation. The following are some of the algorithm's notable contributions:

• Each node connects with the other node and transfers directions of the accurate load sharing and regulation of the node.
• Backward Neural (BNN) network is used at secondary level control to maintain the operation of DC MG during communication breaks and latencies.
• Conventional control has the inherent vulnerability to communication failures or latencies.
• BNN has the adaptive nature which makes its effect for the communication latencies and breaks.

The Paper will follow Section 2 The communication link with Backward Neural Network as shown in the section. Section 3 explains the Neural Network. In Section 4, Backward propagation is explained, whereas Section 5 consists of the case study and its results. Finally, the paper is concluded in Section 6.

## 2 Effects of communication network latencies in DC microgrid (MG)

**Fig. 1:**
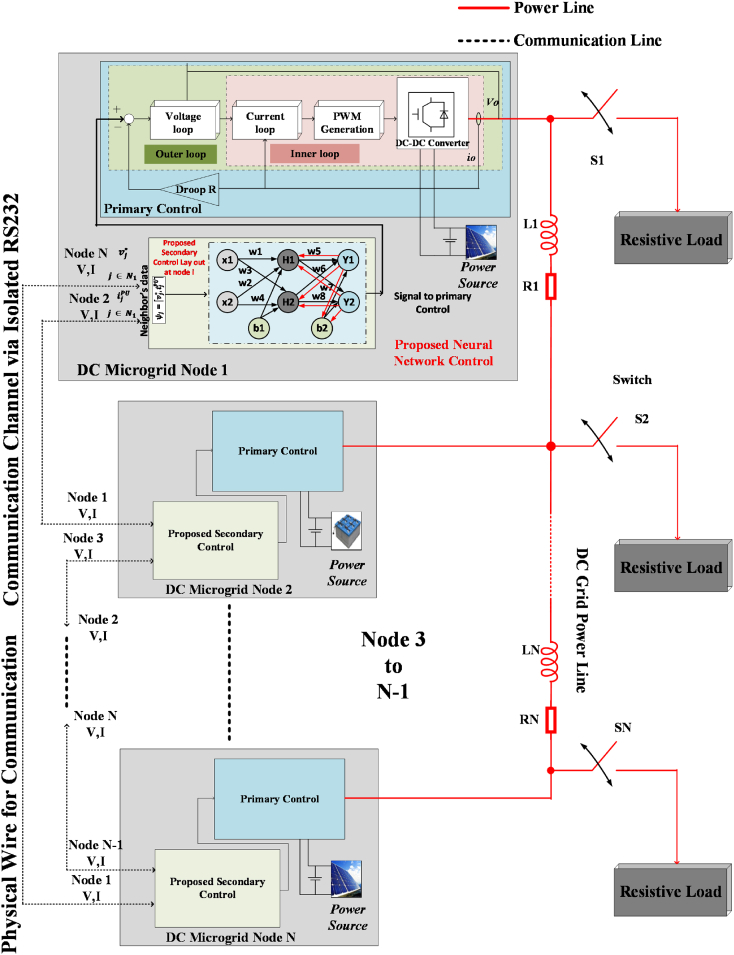
BNN-based proposed secondary control of the DC microgrid (DC MG).

**Fig. 2:**
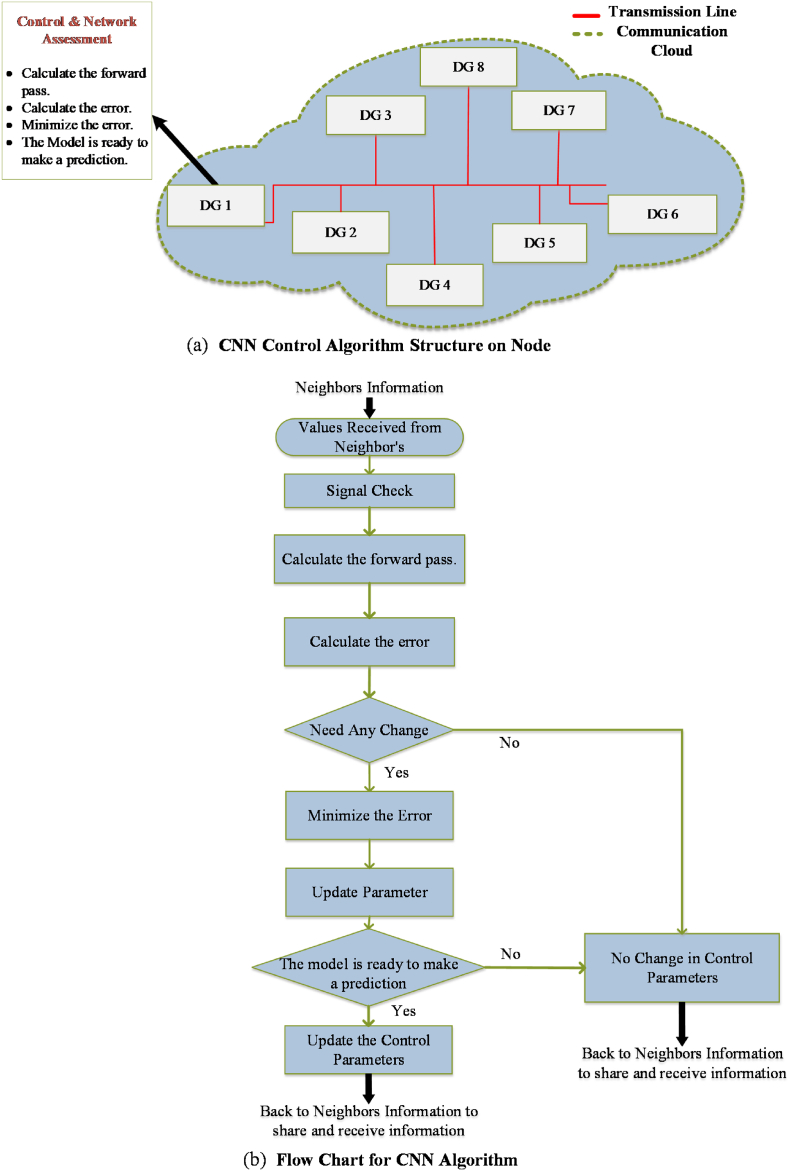
Proposed DC MG: (a) Single node structure of proposed control; (b) Proposed control flow chart.

In BNN-based multi-node system extensively uses the graphical representation to map MG information layer communication. BNN has some countable advantages over simple neural networks i.e., uncertain quantification, robustness to overfitting, handling small datasets, model flexibility, and adaptive learning. These properties make the BNN more attractive in RES-based intermittent nature systems. A sparse network is spread all over the DC MG to share the instruction set points for each node as shown in Fig. 1. The communication network is utilized to share the instruction set points, but in case of communication failure, the set points will deviate from the real set point. The conventional control fails in case of deviated set points. However, BNN has to be trained by a higher amount of data for a more accurate outcome, and with less data it will be difficult to predict the result accurately. A backward neural network (BNN) will back the DC MG control during communication latencies or failures. The communication system may be divided into one or more communication networks. These divided nodes may affect the performance of the MG and may work as a load. As seen in Fig. 1, information about the network access map is shared between agents to accomplish the single operating point. During the faulty condition, the isolated node cloud will use the proposed BNN to maintain its working. On the other hand, all the other linked nodes also used the proposed control to share the information of each node's parameters and converge to a single point of operation [27,57]. In comparison, the operating point for isolated communication nodes and other nodes would be different. This difference will put a strain on the nodes that are connected to the electrical network [58]. To address this issue, the proposed BNN for secondary control will manage the communication network of the DC MG at every node. BNN is quick and reliable for connecting and transferring information as compared to conventional consensus control. The neural network will maintain system health between nodes in case of communication failure or latencies. Fig. 2 (a) and (b) represents the proposed secondary control node structure and flow map which are utilized by each node of DC MG. The implemented node structure is depicted in the secondary control of Fig. 1.

## 3 Neural network (NN)

**Fig. 3:**
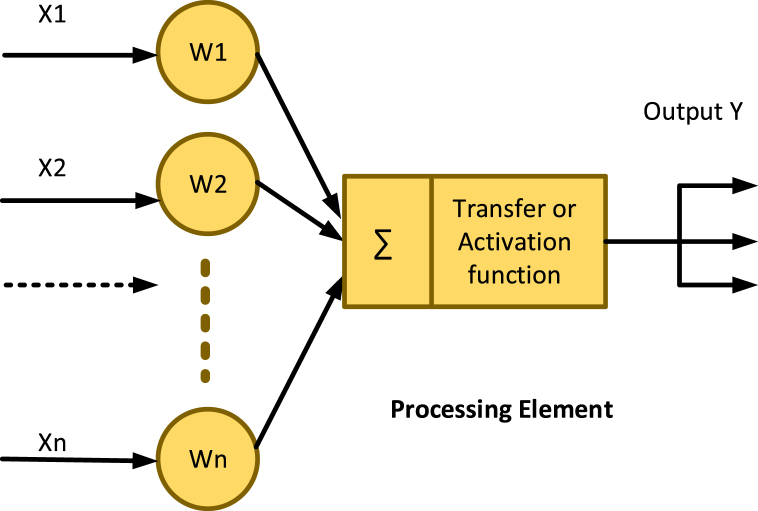
Neural network algorithm.

A neural network (NN) consists of neurons, which make a structure to help in transferring information. Its concept was taken from the biological neural networks. Neural network (NN) also works as a human neural network (if a human's hand touches a hot thing, then neurons give signals to the brain and the brain sends a signal back to the hand to remove or move the hand from the hot surface). In 1943, Pitts and McCulloch presented a Mathematical model of a neural network for the first time, which would not be an easy thing to implement [59]. The neuron model of the neural network is easy to use for building connected networks and sharing information. In the model, synaptic power is represented by weights of neuronal relation [59]. The generalized neural network algorithm model is shown in Fig. 3.

NN depends on nodes, weight, bias, hidden layers, and activation function.

Nodes are the input of neurons like $x_{1},x_{2},\ldots \ldots ..x_{n}$ in the above figure. Weight is like input which multiplies with the input. Mostly weight is represented by w as shown in Eq (1).

(1) $$x_{1}w_{1}+x_{2}w_{2}+\ldots$$

Bias is like input which adds to the input and weight. Mostly bias is represented by b as shown in Eq (2).

(2) $$x_{1}w_{1}+x_{2}w_{2}+\ldots +b$$

**Fig. 4:**
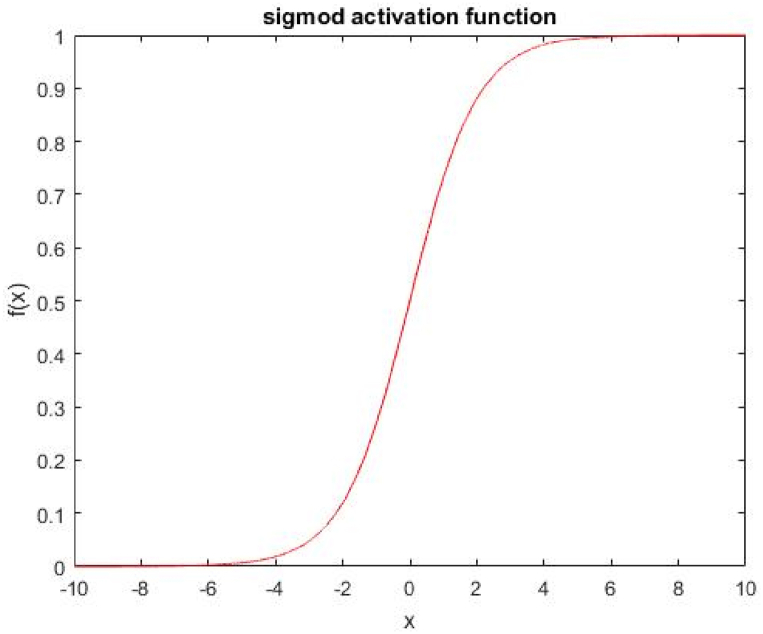
Sigmoid activation function.

The activation function is important because it works as a threshold function, which is a non-linear function [58]. There are many types of the Activation function. The commonly used function is sigmoid as shown in Fig. 4 and Eq (3).

(3) $$f\left(x\right)=1/\left(1+e^{z}\right)$$

NN has different types like backpropagation NN and forward propagation NN. In the proposed work, backpropagation NN is used for secondary control. By using these NN properties secondary control is proposed for robust control during communication latencies.

## 4 Backward neural network (BNN)

The backward neural network algorithm is based on a building block. “Learning representations by backpropagating errors” research work was introduced in 1960 and 30 years later (in 1989) by Williams and Rumelhart, Hinton [60].

The basic idea behind backpropagation is to use the chain rule to compute the gradient of the error by utilizing the weights of the network. The error is defined as the difference between the network's predictions and the true values in the training data, and the gradient is a measure of how much the error changes as the weights are varied. The algorithm works by computing the gradient of the error concerning the outputs of the network, then propagating it back through the network, layer by layer, computing the gradient using the inputs at each layer.

The backpropagation algorithm is typically used in conjunction with an optimization algorithm, such as gradient descent, which uses the gradients computed by backpropagation to adjust the weights of the network in a direction that reduces the error. Several iterations are repeated until the error reaches a satisfactory level or the optimization algorithm converges. Due to these properties and their adjustable behavior, it can withstand latencies and communication delays in the DC MG control structure.

The mathematics behind the BNN is given as [61]:

Eq (4) represents the Input layer,

(4) $$x_{i}=a_{i}^{\left(1\right)},i=1,2,3,\ldots \ldots \text{.}$$

Eq (5) shows the hidden layer, If we consider 2 hidden layers in the system. The first layer of the hidden layer is $l_{1}=2$.

(5) $$H^{\left(2\right)}=W^{\left(1\right)}x+b^{\left(1\right)}$$

(6) $$a^{\left(2\right)}=f\left(z^{\left(2\right)}\right)$$

Eq (6) shows the output of hidden layer which is also used as input for the next hidden layer.

The second layer of the Hidden layers is $l_{1}=3$.

(7) $$H^{\left(3\right)}=W^{\left(2\right)}a^{\left(2\right)}+b^{\left(2\right)}$$

(8) $$a^{\left(3\right)}=f\left(z^{\left(3\right)}\right)$$

where $b^{1}$ from Eq (5) and $b^{2}$ Eq (7) are biased,

$W^{\left(1\right)}$ and $W^{\left(2\right)}$ are weighted given in Eqs (5), (7) and

$a^{\left(2\right)}$ from Eq (6) and $a^{\left(3\right)}$ from Eq (8) are non-linear functions which are also known as the Activation function.

Some operations apply on any layer.

Eq (9) represents the value of $W^{\left(1\right)}$. $W^{\left(1\right)}$ is a Weight matrix (n,m), where n is the number of output and m is the number of input neurons. For n=2 and m=3.

(9) $$W^{1}=\left[\begin{array}{cc} \begin{array}{cc} W_{11}^{1} & W_{12}^{1}\\ W_{21}^{1} & W_{22}^{1} \end{array} & \begin{array}{cc} W_{13}^{1} & W_{14}^{1}\\ W_{23}^{1} & W_{24}^{1} \end{array} \end{array}\right]$$

x is the input vector which is represented in Eq (10), in the matrix form (n,1)

(10) $$x=\left[\begin{array}{c} \begin{array}{c} x_{1}\\ x_{2} \end{array}\\ \begin{array}{c} x_{3}\\ x_{4} \end{array} \end{array}\right]$$

$b^{1}$ is the bias vector as shown in Eq (11), in the matrix form (n,1)

(11) $$b^{1}=\left[\begin{array}{c} b_{1}^{1}\\ b_{2}^{1} \end{array}\right]$$

The equation of $H^{2}$ becomes as shown in Eq (12)

(12) $$H^{2}=\left[\begin{array}{cc} \begin{array}{cc} W_{11}^{1} & W_{12}^{1}\\ W_{21}^{1} & W_{22}^{1} \end{array} & \begin{array}{cc} W_{13}^{1} & W_{14}^{1}\\ W_{23}^{1} & W_{24}^{1} \end{array} \end{array}\right]\;\left[\begin{array}{c} \begin{array}{c} x_{1}\\ x_{2} \end{array}\\ \begin{array}{c} x_{3}\\ x_{4} \end{array} \end{array}\right]+\left[\begin{array}{c} b_{1}^{1}\\ b_{2}^{1} \end{array}\right]$$

(13) $$H^{2}=\left[\begin{array}{c} H_{1}^{2}\\ H_{2}^{2} \end{array}\right]$$

Eq (12) shows $H^{2}$ , which needs a weight matrix, input, and biased. Eq (13) shows the output value on $H^{2}$. Calculate hidden layer 2 in a similar way

Now, the formula of the output layer will be $Y=W^{\left(3\right)}a^{\left(3\right)}$.

Initially, we have target output or expected output T. We would find the output Y and then find (MSE) Mean squared error E. MSE function evaluated between Y and.

According to Ref. [60], BNN repeatedly adjusts the weight values to get or nearest value to the desired output or expected output. BNN minimizes the error function by changing biases and weight values. The level of change is according to the error function w.r.t parameters.

In backpropagation NN, first of all, calculate simply forward pass,

Secondly, calculate the error,

Thirdly, minimize the error,

Fourthly, update the parameters,

Fifthly, the model is ready to make a prediction.

Example of Backpropagation:

Input, $x_{1}=0.05,x_{2}=0.10$.

Bias, $b_{1}=0.35,b_{2}=0.60$.

Target, $T_{1}=0.01,T_{2}=0.99$.

Weights,

$$W_{1}=0.15,W_{2}=0.20,W_{3}=0.25,W_{4}=0.30\text{,}$$

$$W_{5}=0.40,W_{6}=0.45,W_{7}=0.50,W_{8}=0.55\text{,}$$

**Fig. 5:**
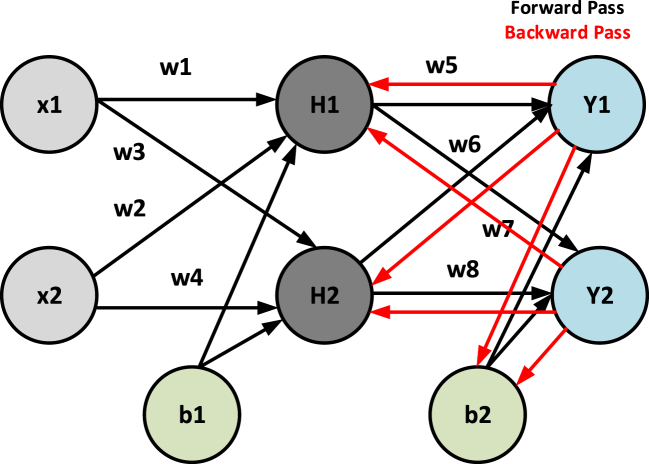
Backward neural network algorithm example.

Fig. 5 shows the backward neural (BNN) network structure by declaring all the variables.

Solution:

Calculates $h_{1}$ in Eq (14) where, $x_{1,}\;x_{2}$ are inputs, $W_{1},W_{2}$ are weights and $b_{1}$ are biased.

(14) $$h_{1}=\left(x_{1}W_{1}+x_{2}W_{2}\right)+b_{1}=0.3750$$

Calculate $H_{1}$ in eq (15) by using $h_{1}$.

(15) $$H_{1}=\frac{1}{1+e^{-h_{1}}}=0.5927$$

Calculates $h_{2}$ in Eq (16) where, $x_{1,}\;x_{2}$ are inputs, $W_{3},W_{4}$ are weights and $b_{1}$ are biased

(16) $$h_{2}=\left(x_{1}W_{3}+x_{2}W_{4}\right)+b_{1}=0.6425$$

Calculates $H_{2}$ in eq (17) by using $h_{2}$.

(17) $$H_{2}=\frac{1}{1+e^{-h_{2}}}=0.6553$$

Calculates $y_{1}$ in Eq (18) where, $H_{1},H_{2}$ are inputs, $W_{5},W_{6}$ are weights and $b_{2}$ are biased.

(18) $$y_{1}=\left(H_{1}W_{5}+H_{2}W_{6}\right)+b_{2}=1.1320$$

Calculate $Y_{1}$ in eq (19) by using $y_{1}$.

(19) $$Y_{1}=\frac{1}{1+e^{-y_{1}}}=0.7562$$

Calculates $y_{2}$ in Eq (20) where, $H_{1},H_{2}$ are inputs, $W_{7}W_{8}$ are weights and $b_{2}$ are biased.

(20) $$y_{2}=\left(H_{1}W_{7}+H_{2}W_{8}\right)+b_{2}=1.2240$$

Calculate $Y_{2}$ in eq (21) by using $y_{2}$.

(21) $$Y_{2}=\frac{1}{1+e^{-y_{2}}}=0.7728$$

E is an error in calculation as shown in Eqs (22), (23),

(22) $$E=\sum \frac{1}{2}{\left(Target\;value-output\right)}^{2}$$

(23) $$E=E_{1}+E_{2}$$

where $E_{1}\;and\;E_{2}$ are calculated to calculate the total error E as shown in Eqs (24), (25), (26),

(24) $$E_{1}=\frac{1}{2}{\left(T_{1}-Y_{1}\right)}^{2}=0.2784$$

(25) $$E_{2}=\frac{1}{2}{\left(T_{2}-Y_{2}\right)}^{2}=0.0236$$

(26) E=0.2548

Backward Pass:

Consider $w_{5}$,

In Eq (27) Error at $w_{5}$,

(27) $$\frac{\partial E}{\partial w_{5}}=\frac{\partial E}{\partial Y_{1}}*\frac{\partial Y_{1}}{\partial y_{1}}*\frac{\partial y_{1}}{\partial w_{5}}$$

Firstly calculate $\frac{\partial E}{\partial Y_{1}}$ in Eq (28)

(28) $$\frac{\partial E}{\partial Y_{1}}=0.74136507$$

Secondly, calculate $\frac{\partial Y_{1}}{\partial y_{1}}$ in Eq (29)

(29) $$\frac{\partial Y_{1}}{\partial y_{1}}=0.186815602$$

Finally, calculate $\frac{\partial y_{1}}{\partial w_{5}}$ in Eq (30)

(30) $$\frac{\partial y_{1}}{\partial w_{5}}=0.593269992$$

Putting value of $\frac{\partial E}{\partial Y_{1}},\frac{\partial Y_{1}}{\partial y_{1}}$ and $\frac{\partial y_{1}}{\partial w_{5}}$ in Eq (27), it becomes (31)

(31) $$\frac{\partial E}{\partial w_{5}}=0.082167041$$

Updating $w_{5}$ as shown in Eq (32),

(32) $$w_{5}=\eta \frac{\partial E}{\partial w_{5}}\;\left(\text{Where}\;\eta =0.5\;\text{is}\;\text{the}\;\text{learning}\;\text{rate},u\;\text{can}\;\text{choose}\;\text{yourself}\right)$$

Putting the value of $\frac{\partial E}{\partial w_{5}}$ and η in Eq (32), its value in Eq (33),

(33) $$w_{5}=0.4-\left(0.5*0.08216704\right)=0.35891648$$

In the same way, the value of $w_{6},w_{7}\text{,}$ and $w_{8}$ will be calculated as shown in Eqs (34), (35), (36),

(34) $$w_{6}=0.408666186$$

(35) $$w_{7}=0.511301270$$

(36) $$w_{8}=0.061370121$$

Now at the hidden layer updating $w_{1}$ by utilizing Eq (27), (28), (29), (30), (31), (32), (33). The updated steps are shown in Eq (37), (38), (39), (40), (41), (42), (43), (44), (45), (46), (47), (48), (49), (50), (51), (52),

(37) $$\frac{\partial E}{\partial w_{1}}=\frac{\partial E}{\partial H_{1}}*\frac{\partial H_{1}}{\partial h_{1}}*\frac{\partial h_{1}}{\partial w_{1}}$$

(38) $$\frac{\partial E_{1}}{\partial H_{1}}=\frac{\partial E_{1}}{\partial y_{1}}*\frac{\partial y_{1}}{\partial H_{1}}$$

(39) $$\frac{\partial E_{1}}{\partial y_{1}}=\frac{\partial E_{1}}{\partial Y_{1}}*\frac{\partial Y_{1}}{\partial y_{1}}$$

(40) $$\frac{\partial E_{1}}{\partial y_{1}}=0.138498562$$

(41) $$\frac{\partial y_{1}}{\partial H_{1}}=w5=0.40$$

(42) $$\frac{\partial E_{1}}{\partial H_{1}}=0.130498562*0.40=0.055399425$$

(43) $$\frac{\partial E_{2}}{\partial H_{2}}=-0.019049119$$

(44) $$\frac{\partial E}{\partial H_{1}}=0.055399425+\left(-0.019049119\right)=0.03635$$

(45) $$H_{1}=\frac{1}{1+e^{-h1}}$$

(46) $$\frac{\partial H_{1}}{\partial h_{1}}=H_{1}\left(1-H_{1}\right)$$

(47) =0.5932(1−0.5932)=0.241300709

(48) $$h_{1}=w_{1}x_{1}+w_{2}x_{2}+b_{1}$$

(49) $$\frac{\partial h_{1}}{\partial w_{1}}=x_{1}=0.05$$

Put values in Eq (37),

(50) $$\frac{\partial E}{\partial w_{1}}=0.03635*0.241300709*0.05=0.00438668$$

Updating w1:

(51) $$w_{1}=w_{1}-\eta \frac{\partial E}{\partial w_{1}}$$

(52) $$w_{1}=0.149780716$$

After performing the calculations for $w_{1}$, the same process is repeated for $w_{2}$, $w_{3}$ and $w_{4}$ using equations (37), (38), (39), (40), (41), (42), (43), (44), (45), (46), (47), (48), (49), (50), (51), (52) as shown in Eqs (53), (54), (55),

(53) $$w_{2}=0.19956143$$

(54) $$w_{3}=0.24975114$$

(55) $$w_{4}=0.29950229$$

Again, calculate the forward pass till achieving the target value and with each iteration the accurse improves. A generalized example is used to express the BNN working.

## 5 Case study

**Fig. 6:**
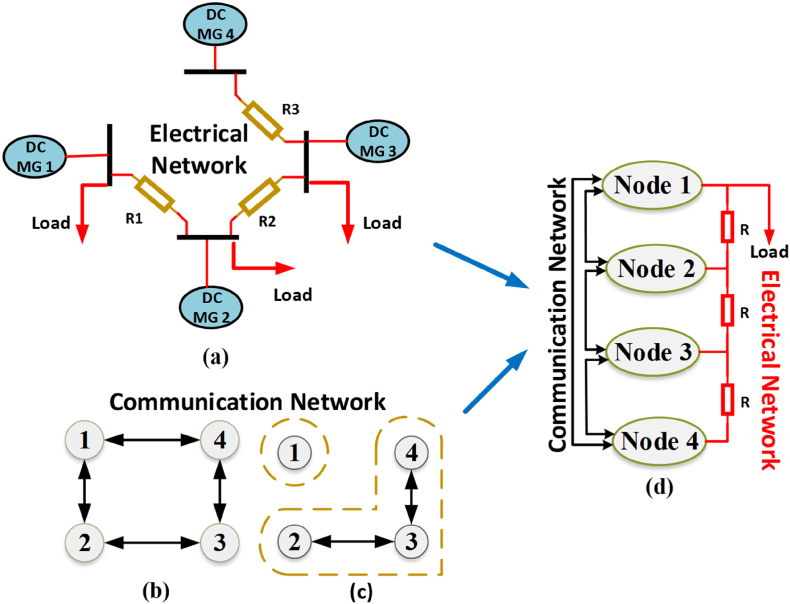
Case study scenario for DC MG network.

A four-node DC microgrid (MG) setup is considered for checking the effectiveness of the proposed BNN control. For the power requirement, each node depends on the renewable energy system and is connected in the radial form. A radial connection is used for sharing the energy. A bi-directional communication configuration in ring form is used to evaluate the effectiveness and functioning of the proposed neural network-based control system. RS232 was used for the communication between nodes to exchange reference values. Communication delays were also considered for the real-time effect of the case study setup. A comprehensive switch model is utilized for the node power converters for an accurate outcome. A two-way circular communication structure is used to share reference value with its neighbors as depicted in Fig. 6 (b)(c). The case study scenario is depicted in Fig. 1, Fig. 6 (a)(d), which is based on the mathematical calculation provided in the above section. Fig. 6 (d) shows the exact scenario taken for the simulating study and line impedance is also selected in the case study for its effect. Node parameter details are presented in Table 1 [53].

**Table 1:** Single node parameters of the case study DC MG structure.

Parameters	Values
V in (Voltage at Input)	600V
Vo (Voltage at Output)	400V
Resistive Load	40 Ω
Resistive Load for load variation test	80 Ω
$G_{\text{Droop}}$ (Droop Gain)	0.025
Resistance Line	0.0005 Ω/m
Inductance Line	0.50 μH/m
Length Line	100 m
Switching Frequency	10 kHz
Voltage Observer	Kp = 6 Ki = 0.1
Filter Inductor	1 mH
Filter Capacitor	300 μF
Channel Bandwidth (Communication)	0.15 m s
Internal Current Loop	Kp = 10 Ki = 0.05
External Voltage Loop	Kp = 40 Ki = 0.05
Current Controller	Kp = 0.11 Ki = 0.6

A backward neural network (BNN) is used for secondary control of the DC MG setup. BNN control uses the value case study values for the DC MG operation and it transfers information to all nodes. S2 switch was considered in the case study, a single node is connected with the DC MG network for the worst-case scenario. S2 is used to add or drop the load from the DC MG for the load variation test. In case of any communication link failure, BNN helps to maintain its working. BNN is a robust control algorithm for communication link failure scenarios. With the help of BNN, DC MG maintains the stability of the DC microgrid. During normal conditions, every neighbor node communicates with each other. Every node generates its energy using renewable energy [62]. The generated energy is utilized using the primary control and secondary control. BNN is applied on secondary-level control using the input from neighbors’ nodes. Submission of these inputs multiplies with weight values and adds bias. This value goes into the sigmoid activation function. This value is called input ($input\;x=x_{i}w_{i}+b$) to the hidden layer. Similarly, hidden values work as input to the output layer on every node. This algorithm makes the system stable in case of any communication link failure. This algorithm improves the robustness of the system during the link failure. When traditional secondary control meets with communication link failure then the system does not maintain its stability and does not continue working. Due to a mismatch in the reference value, the DC MG system becomes unstable and this situation may lead the microgrid to cascaded failure.

**Fig. 7:**
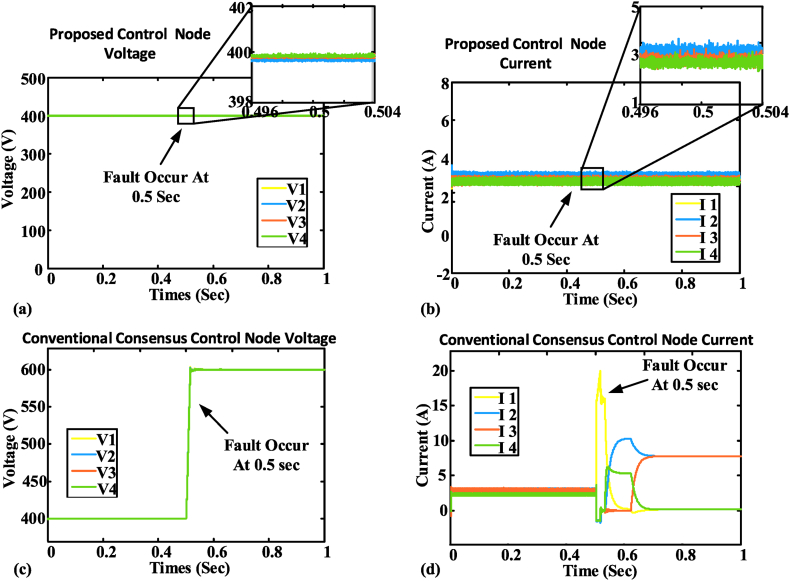
The outcome of the case study network: (a) Node voltage of BNN control; (b) Node current of BNN control; (c) Node voltage of existing control; (d) Node current of existing control.

**Fig. 8:**
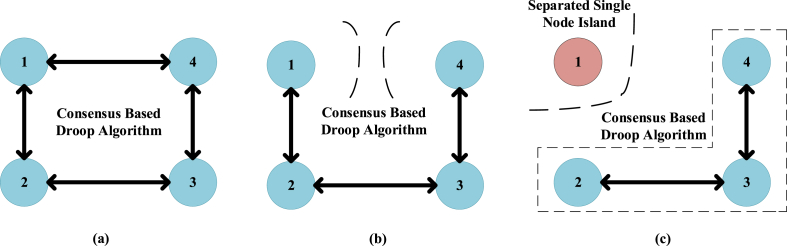
Different scenarios for DC MG.

Accordingly, the microgrid current sharing and output voltage remain stable for the proposed control during the communication link failure [11,63]. As depicted in Fig. 7(a), the proposed control voltage remains stable and operational as the communication fault occurs at 0.5 s. As in Fig. 7 (b), the proposed control current also remains stable during communication failure as a fault occurs at 0.5 s. Whereas, the conventional control becomes unstable during the communication link failure. A communication failure problem occurs in the DC MG at 0.5 s as shown in Fig. 7(c) the conventional control voltage becomes unstable and starts to follow input as output at the time of fault occurrence. As in Fig. 7 (d), the conventional control current also becomes unstable during the communication fault occurs at 0.5 s, due to the mismatch in the reference values. The graphical illustration of the DC MG is depicted in Fig. 8 for different states. Fig. 6, Fig. 8 shows the communication fault occurrence in the case study simulation. Where one node is disconnected from the network and forms its communication islands. In this case, the proposed control shows robustness against this issue. Nevertheless, traditional control systems are unable to cope with the communication island, resulting in the loss of the reference point and the system starting to track the input as the output. The communication fault occurs at 0.5 s as depicted in Fig. 7. A two-way communication system is considered to have a balanced Laplacian matrix for communication connectivity using $a_{ij}$ to produce correction terms. Fig. 8(a) depicts the total connected nodes in the form of mathematical form as presented in Equation (56):

(56) $$L=\left[\begin{array}{cc} 2-1-12 & 0-1-10\\ 0-1-10 & 2-1-12 \end{array}\right]$$

In the same way, Fig. 8(b) depicts a single communication failure that disturbs the connected network. After the single link failure, the Laplacian matrix will be changed as presented in Equation (57). Then again, if both links of node 1 fail, as shown in Fig. 8(c), it's Laplacian for the remaining connected nodes will be turned into Equation (58):

(57) $$L=\left[\begin{array}{cc} 1-1-12 & 00-10\\ \begin{array}{cc} 0 & -1\\ 0 & 0 \end{array} & 2-1-11 \end{array}\right]$$

(58) $$L=\left[\begin{array}{ccc} 1 & -1 & 0\\ -1 & 2 & -1\\ 0 & -1 & 1 \end{array}\right]$$

**Fig. 9:**
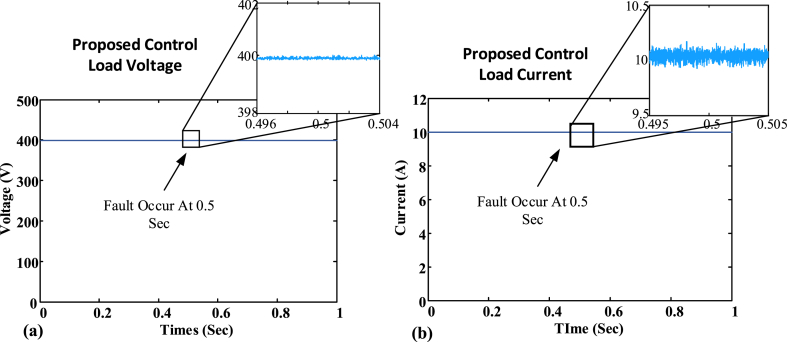
DC MG output: (a) Load voltage; (b) load current.

**Fig. 10:**
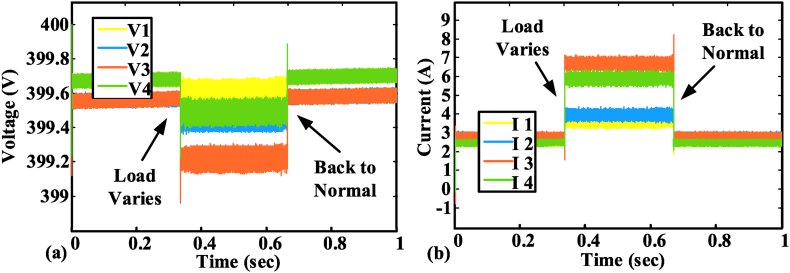
Load variation test on the case study setup: (a) Node voltage; (b) node current.

Fig. 9 (a) and (b) illustrates the load voltage and current to demonstrate the efficacy of the control on the load. The load-sharing and voltage regulation efforts among all nodes are sustained by the proposed control. The proposed BNN control maintains DC MG operation and working. Whereas the fault occurs at 0.5 s, and the BNN control detects the faults and maintains the working of the DC microgrid. Plug-and-play properties are observed in the BNN control structure. BNN control can easily adjust the DC microgrid node variation. The suggested control system in Fig. 8 can remain operational even if one or more nodes are added or withdrawn. The proposed BNN control has been tested for the plug-and-play operation. A load variation test has been conducted on the BNN control and it performs the DC microgrid (MG) operation smoothly as shown in Fig. 10 (a) and (b). Fig. 10 (a) and (b) show that at the time of an increase in load, a minor decrease in voltage occurs but it remains in the safe operating region. On the other hand, the current in the figure increases to meet the load requirement without disturbing the DC microgrid (MG) operation. A detailed comparison is given in Table 2 and Table 3.

**Table 2:** Comparison of the proposed BNN control with an existing control structure.

Sr. No	Parameters	Conventional Consensus [[64], [65], [66]]	Distributed Secondary Voltage and Frequency Control [67]	Proposed Control
**1**	Single Point of Failure	No	No	No
**2**	Communication Network	Yes	Yes	Yes
**3**	Mismatch in Reference Values	No	No	No
**4**	Simplicity in Control Structure	Complex Structure	Complex AC Network	Complex Structure
**5**	Converge MG to Global Reference Points	Yes	Yes	Yes
**6**	Detection of Link Failure	No	No	No
**7**	Converge MG to Global Reference Points During Link Failure	No	No	Yes
**8**	Detection of Islands Formation and Maintain System Operation	No	No	Yes

**Table 3:** Comparison of a secondary control system for conventional control and proposed control.

Comparison table of Case study Scenario					
Cases		Conventional system		Proposed system	
		Input	Output	Input	Output
**Timing: 0 to 0.5**	Normal system operation	Reference value from nodes	Expected output to maintain working	Reference value from nodes	Expected output to maintain working
		A conventional system takes an input value of 400 for $v_{1}$ , 400 for $v_{2}$ and 400 for $v_{4}$ and gives 0 in output. The conventional system maintains its stability, working, and operation in a normal situation.		A normal system takes an input value of 0.4 and gives 0.9933 in output. The proposed system also maintains the system in a stable situation. It maintains its stability, work, and operation. In a normal situation, the proposed system gives better results than the conventional system.	
**Timing: 0.5 to 1**	In case of link failure	Reference value failure	Generates error value and the system becomes unstable	Reference value failure	Neglecting error value and maintaining system
		In case of link failure, take an input value of 400 for $v_{1}$ , 0 for $v_{2}$ and 400 for $v_{4}$ and give 0 in output at time −400. The conventional system becomes unstable due to link failure. It does not maintain its stability, working, and operation.		When a link failure occurs, the system takes the input value 0 for v and gives 0 in output. The proposed system does not become unstable due to link failure. It shows robustness for its stability, work, and operation in case of a link failure situation.	

## 6 Conclusion

This study proposed a new control technique for DC microgrids (MG) that uses a backward neural network (BNN). The goal is to balance load distribution while regulating voltage, without compromising accuracy, even when communication links fail. BNN achieves this by sharing operation directions between nodes using a communication network. When communication breaks or latencies occur, BNN maintains the operating direction at each node based on the last shared direction. Other connected nodes continue to operate and share the direction for accurate load distribution and voltage regulation. This proposed BNN algorithm scheme is more robust and adaptable in comparison to conventional controls that cannot withstand communication breaks or latencies. Detailed mathematical analysis and comparison with conventional control have verified the usefulness of this proposed strategy. However, BNN requires a larger amount of data for more accurate outcomes, and less data can make it difficult to predict the result accurately. In the future, this proposed BNN control can be extended to multi-feeders with a higher degree of complex connectivity in the communication and electrical networks of the DC microgrid due to its adaptive nature.

## Data availability statement

Data Will be made available on request from authors.

## CRediT authorship contribution statement

**Hira Anum:** Writing – original draft, Software, Methodology, Conceptualization. **Muntazim Abbas Hashmi:** Writing – original draft, Software, Methodology, Investigation, Data curation. **Muhammad Umair Shahid:** Software, Project administration, Formal analysis, Data curation, Conceptualization. **Hafiz Mudassir Munir:** Visualization, Validation, Software, Resources, Conceptualization. **Muhammad Irfan:** Writing – review & editing, Methodology, Investigation. **Veerendra A.S:** Writing – review & editing, Funding acquisition, Formal analysis. **Mohammad Kanan:** Writing – review & editing, Validation, Funding acquisition. **Aymen Flah:** Writing – review & editing, Supervision.

## Declaration of competing interest

The authors declare that they have no known competing financial interests or personal relationships that could have appeared to influence the work reported in this paper.
